# Statistical analysis of mental influencing factors for anxiety and depression of rural and urban freshmen

**DOI:** 10.3389/fpubh.2023.1235262

**Published:** 2023-12-22

**Authors:** Chang Li, Bingchuan Sun

**Affiliations:** ^1^College of Insurance, Shandong University of Finance and Economics, Jinan, China; ^2^College of Physical Education, Shandong University of Finance and Economics, Jinan, China

**Keywords:** PPS investigation, anxiety, depression, canonical correlation analysis, path analysis, education

## Abstract

The freshmen stage is a high incidence period for psychological issues. With the increasing gap between urban and rural areas in China, the mental problems of rural freshmen are more prominent in recent years due to the huge contrast of campus life with their growth environment and other reasons. The concern for the mental well-being of both rural and urban freshman students prompted our comprehensive five-year study (2018–2022) on psychological issues in a group of 12,564 first-year students from dozens of public universities in Shandong province. The investigation employed PPS (probability proportional to size) sampling and was conducted near the the end of the first semester. Using the data gathered, we analyzed and compared the indicators of psychological problems in rural and urban freshmen by Duncan's Multiple Range Test. We also conducted a canonical correlation analysis and pathway analysis to examine the psychological factors that contribute to anxiety and depression in both rural and urban freshmen. According to the findings, rural freshmen exhibit significantly higher levels of anxiety and depression than their urban counterparts. Inferiority, obsession, and internet addiction were identified as the primary influencing factors of anxiety and depression in both rural and urban freshmen. Social phobia was found to be a significant influencing factor for anxiety in rural freshmen, while bigotry was identified as a specific influencing factor for urban freshmen. Furthermore, the results of the path analysis suggest that anxiety plays a crucial role as a mediating factor between the main influencing factors and depression. These results substantially extend former research in this area and have important implications for the development of effective intervention strategies to address anxiety and depression. According to these results, policymakers should assess and intervene of anxiety and depression as a whole, and provide mental health education according to main effect factors of freshmen from rural and urban areas. Detailed policy recommendations are in discussion and conclusion.

## 1 Introduction

Previous research has highlighted a high prevalence of mental health problems, specifically depression and anxiety, among undergraduate students (1–5). Adlaf et al. (6) pointed out for college students, there is a prominent inverse relationship between year of study and mental health. Lee et al. (7) studied stress, anxiety, and depression symptoms for students in a public research university in Kentucky during an early phase of COVID-19, and found rural, low-income, and academically underperforming students were more vulnerable to these mental health issues. Amir Hamzah et al. (8) studied the prevalence and related factors of depression and anxiety of freshmen in a learning institution in Malaysia, and found students lived with non-family members were more likely to have depression and anxiety.

Some recent research about the anxiety and depression include: Liu et al. (9) investigated the longitudinal relationship between anxiety and self-esteem among college students, and confirmed self-esteem as one of the leading contributors to anxiety for college students. Liu et al. (10) reviewed the literature on risk factors and digital interventions for college students' anxiety disorders from the perspectives of different stakeholders, emphasizing the important roles played by different stakeholder groups, and provides valuable references for improving the mental health of college students. Liu et al. (11) reviewed the extant literature by identifying non-pathological factors related to college students' depression, investigating the methods of predicting depression, and exploring non-pharmaceutical interventions for college students' depression.

In China, the urban-rural gap, such as income ratio, is one of the highest in the world, and the urbanization in recent years has widened the gap between urban and rural development (12–14). Thus freshmen from rural area are more likely to have serious mental problems due to the huge contrast of campus life with their growth environment and other reasons (15–17). Some typical research about mental health of rural and urban freshmen in China include: Yulong (18) and Zhixue (19) conducted comparative analysis for the mental health of undergraduate students from rural and urban areas, and found the psychological health level of rural freshmen is much lower than and urban freshmen. Yuemin (15) and Zhang (20) investigated and analyzed the mental health of college freshmen, and found the psychological problems of rural freshmen are more serious than urban freshmen. Other related research include: Wang and Wang (21) and Wenting and Zhiqiang (22), etc.

Previous research has established significant theoretical and practical foundations for studying the mental health issues of freshmen. Nonetheless, previous studies have rarely conducted comprehensive investigations into the mental health problems of freshmen from both rural and urban areas in specific provinces in China, nor have they conducted systematic statistical analyses of the factors influencing anxiety and depression among freshmen.

In this research, we took sampling investigation and statistical analysis for mental problems of totally 12,564 freshmen in dozens of public universities in Shandong province over the past 5 years (2018–2022). Based on these data, we analyzed the mental influencing factors of depression and anxiety for rural and urban freshmen separately by canonical correlation analysis and path analysis. Our findings have significant implications for the development of practical intervention strategies and the promotion of mental health among college students.

## 2 Materials and methods

SAS 9.4, R 4.1.2 and Mplus 7 were used for data analysis. The computer code used are available upon request.

### 2.1 Design of investigation

In each of the past 5 years, we have taken PPS (probability proportional to the population size sampling) investigations to freshmen in dozens of public universities in Shandong province near the end of their first semester (usually between late November through early December). The main reason that we choose this investigation period is: the freshmen in China usually have military training in the first month of their first semester, and they need a few month to adapt to college life. On the other hand, the final exams in China usually begin at the end of December or early January, thus the mental status of freshmen is relatively stable in our investigation time period.

The minimum sample size *N* for each year is determined by Ross (23) and Wackerly et al. (24):


(1) $$\begin{array}{l} N=\frac{Z_{\alpha }^{2}p\left(1-p\right)}{E^{2}} \end{array}$$

Under 95% confidence level (thus $Z_{\alpha }^{2}=1.96$), we set *p* = 0.5 (the most conservative method), error rate E = 2%, and get *N* = 2,401. Thus in each year, around 3,000 students were chosen. The participants were chosen randomly by probability sampling, and investigations were sent to them by Enterprise WeChat. Each university's sample size is proportional to the freshmen enrolled in.

The main scale of the investigation is the mental health screening scale for Chinese college students In addition to the demographic characteristics of the participants, the scale includes 22 indicators related to mental problems: anxiety, depression, bigotry, inferiority, sensitive, social phobia, somatization, dependency, hostile attack, impulsion, obsession, internet addiction, self injurious behavior, eating problems, sleep disturbance, university adaptation difficulties, interpersonal troubles, academic pressure, employment pressure, trouble in courtship, suicidal intent, hallucinations, and delusions. The participants' demographic characteristics and descriptive statistics for mental problems indicators are in Section 3.1. Each indicator's score is represented by the index standard score (Z-score), which correlates with the level of severity of the mental issue. Fang et al. (25) have introduced the development of this scale and confirmed the reliability and the validity of the scale all reached the criterions of psychological assessment.

Before commencing the questionnaire, the participants provided written informed consent online. The research procedures adhered to the American Association for Public Opinion Research (AAPOR) reporting guidelines and were approved by the Research Ethics Committee at Shandong University of Finance and Economics in China.

### 2.2 Comparison of mental problems of freshmen from urban and rural areas

We use Duncan's multiple range test to conduct comparison of anxiety, depression, and other important indicators of mental problems of freshmen from urban and rural areas. Due to the limited space, we only show the contrast of anxiety and depression in Section 3.2. The comparison of other indicators of mental problems are available upon request.

The contrast of anxiety and depression of for rural and urban freshmen is in **Tables 3**, **4**. The results show the means of anxiety and depression of rural freshmen are significantly higher than that of urban freshmen (detailed analysis is in Section 3.2). Thus we should analyze the mental effect factors for anxiety and depression separately for rural and urban freshmen.

### 2.3 Statistical analysis for mental effect factors of anxiety and depression

Previous research suggests that anxiety and depression often comorbid, and the comorbidity of these two conditions is a relatively frequent syndrome (26–29). Therefore, we considered anxiety and depression as a whole, and processed by canonical correlation analysis.

Canonical correlation analysis is a statistical technique used to explore the relationship between two sets of variables. It helps identify and measure the associations between two multivariate data sets, seeking linear combinations of variables in each set that have the highest correlation with each other.

The statistical analysis involved three steps: firstly, we identified the significant influencing factors of anxiety and depression separately using linear regression for rural and urban freshmen. Then, we performed canonical correlation analysis on the significant influencing factors (independent variables) and anxiety and depression (dependent variables). The core principle of canonical correlation analysis is to transform the correlation between multiple variables into the correlation between two representative variables (30–33). In this study, we used the linear combination of anxiety and depression as one representative variable, and the linear combination of the significant influencing factors as another representative variable. The results of this analysis are presented in Section 3.3.

After that, based on the results of canonical correlation analysis, we conducted path analysis for the mediating effect of anxiety on the relationship between the effect factors and depression.

### 2.4 Path analysis

Since the effect factors of anxiety and depression are multiple mental index of the same population, we process path analysis with multiple independent variables (path analysis with each single independent variable are available upon request).

Path analysis is a statistical method for examining relationships between variables to understand how they influence one another. It helps researchers understand complex causal relationships by analyzing direct and indirect effects among variables. The principle, models and methods of path analysis with multiple independent variables are introduced in Chapter 10 of (34), Chapter 9 of (35). Based these models and methods, we construct the path analysis on below.

By the results in canonical correlation analysis in Section 3.3, for rural freshmen, the top 4 influencing factors for anxiety and depression are inferiority, obsession, somatization, and social phobia. For urban freshmen, the top 4 effect factors are inferiority, obsession, somatization, and bigotry. In path analysis, we take the top 4 effect factors for anxiety and depression in canonical correlation analysis and internet addiction as independent variables. Internet addiction is added in path analysis because the effect of internet addiction on anxiety and depression in recent years have attracted widespread concern in previous research (36–38), etc.

The mediation effect of anxiety on the relationship between the effect factors and depression is studied in several previous works, such as Cummings et al. (29), Moscovitch et al. (39), Nima et al. (40), etc. For example, Moscovitch et al. (39) pointed out that the intervention reduced anxiety was responsible for 91% of the reduction in depression. Nima et al. (40) studied mediation effect of anxiety on the relationship between stress, self-esteem and depression. In the path analysis, for both urban and rural freshmen, we take depression as the dependent variable, and anxiety as the mediating variable. The results are in Section 3.4.

## 3 Results

All original program results are in figures for reference.

### 3.1 Descriptive statistics for the investigation

In this investigation, only questionnaires with all questions related to demographic characteristics and mental problems answered are considered as valid questionnaires. The basic sociodemographic characteristics of the participants, such as year of investigation, gender and region are in Table 1. In this table, total is the total number of valid questionnaires collected in each year.

**Table 1:** Sociodemographic characteristics of the participants.

**Year**	**Total**	**Male**	**Female**	**Rural area**	**Urban area**
Year 2018	2,502	1,185	1,317	1,006	1,496
Year 2019	2,455	1,098	1,357	978	1,477
Year 2020	2,484	1,135	1,349	1,022	1,462
Year 2021	2,441	1,204	1,237	967	1,474
Year 2022	2,682	1,286	1,396	1,078	1,604

**Figure 1:**
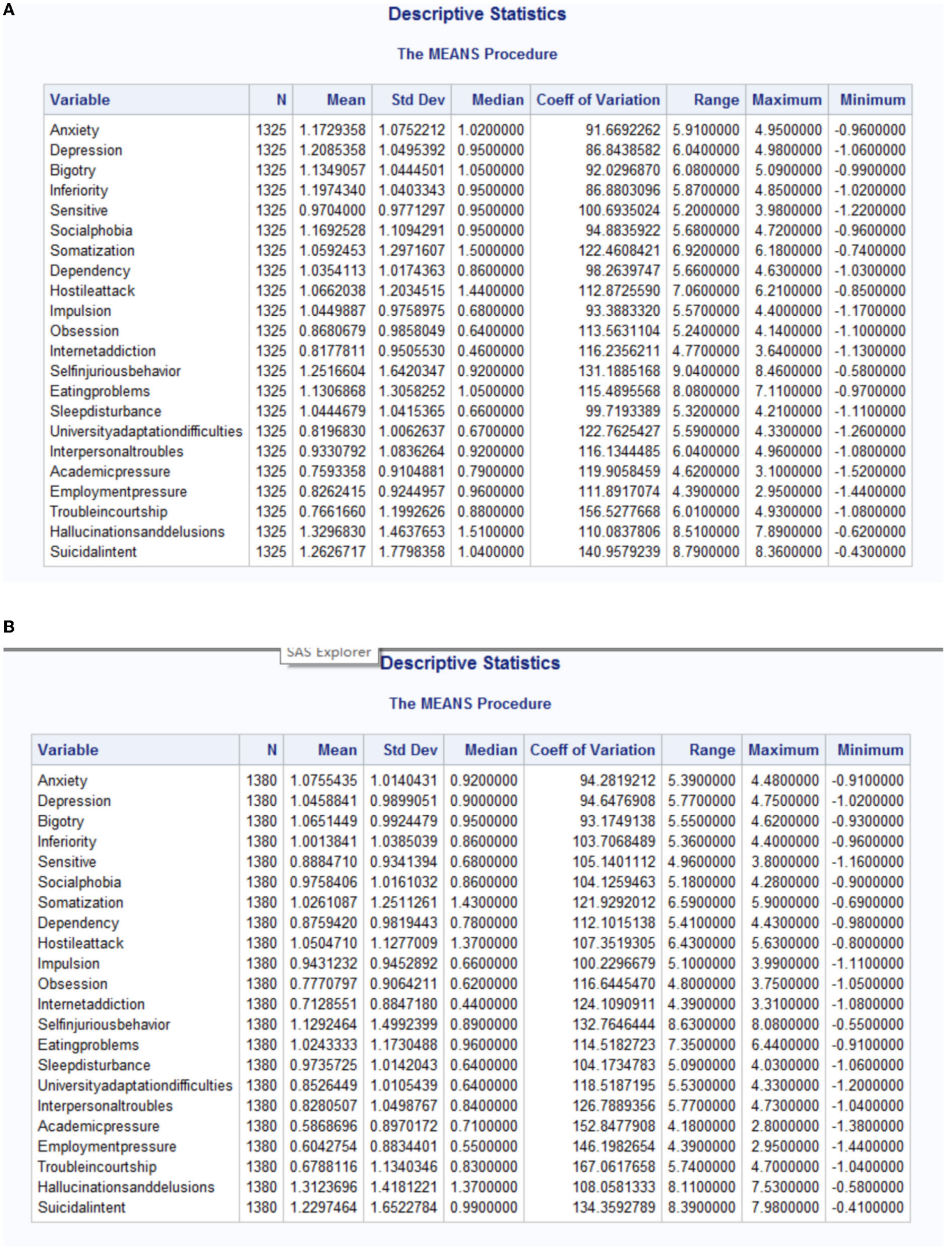
The descriptive statistics for psychological problems in freshmen coming from rural and urban area. **(A)** Descriptive statistics for mental problems of rural freshmen. **(B)** Descriptive statistics for mental problems of urban freshmen.

Totally 1,325 rural freshmen and 1,380 urban freshmen are detected with mental problems. By Table 1, the total valid questionnaires from rural and urban freshmen are 5,051 and 7,513 respectively, thus the proportion of mental problems of rural freshmen is much higher than that of urban freshmen. The descriptive statistics of main indicators is in Table 2. The descriptive statistics for all indicators is in Figure 1 in program results.

**Table 2:** Descriptive statistics for main indicators of rural and urban freshmen.

	**Rural**	**Rural**	**Urban**	**Urban**
**Indicator**	**Mean**	**Standard deviation**	**Mean**	**Standard deviation**
Anxiety	1.173	1.075	1.075	1.014
Depression	1.208	1.049	1.046	0.990
Inferiority	1.197	1.040	1.001	1.038
Obsession	0.868	0.984	0.777	0.906
Somatization	1.059	1.297	1.026	1.251
Internet addiction	0.818	0.950	0.712	0.885

### 3.2 Contrast of anxiety and depression for rural and urban freshmen

**Figure 2:**
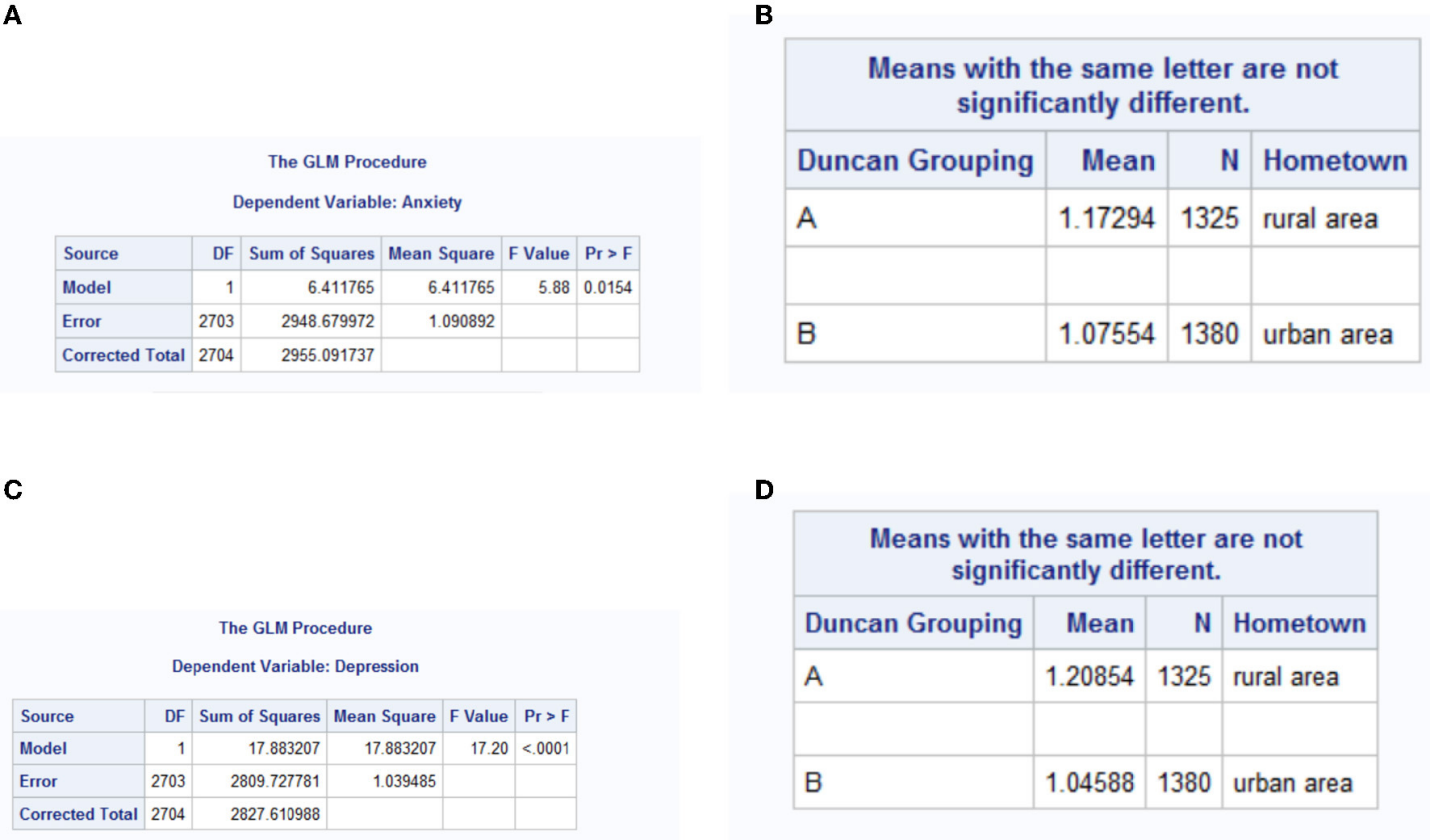
Comparison of anxiety and depression for urban and rural freshmen. **(A)** *P* value of anxiety comparison. **(B)** Means of anxiety. **(C)** *P* value of depression comparison. **(D)** Means of depression.

The contrast of anxiety of for rural and urban freshmen is in Tables 3, 4, and the original program results are in Figure 2. In Duncan's multiple range test, means with different letters are significantly different.

**Table 3:** Comparison of anxiety for urban and rural freshmen.

**Duncan grouping**	**Mean**	**N**	**Hometown**
A	1.17294	1,325	Rural area
B	1.07554	1,380	Urban area

**Table 4:** Comparison of depression for urban and rural freshmen.

**Duncan grouping**	**Mean**	**N**	**Hometown**
A	1.20854	1,325	Rural area
B	1.04588	1,380	Urban area

From the results in Figures 2A, B, the *P* value for comparison of anxiety of rural and urban freshmen is 0.0154. From Table 3, the mean of anxiety for rural freshmen is 1.17294, and marked as A. The mean of anxiety for urban freshmen is 1.07554, and marked as B. From the results in Figure 2C, the *P* value for comparison of depression of rural and urban freshmen < 0.0001. From Table 3, the mean of depression for rural freshmen is 1.20854, and marked as A. The mean of depression for urban freshmen is 1.04588, and marked as B. These results shows the means of anxiety and depression of rural freshmen are significantly higher than that of urban freshmen.

### 3.3 The results for linear regression and canonical correlation analysis

**Figure 3:**
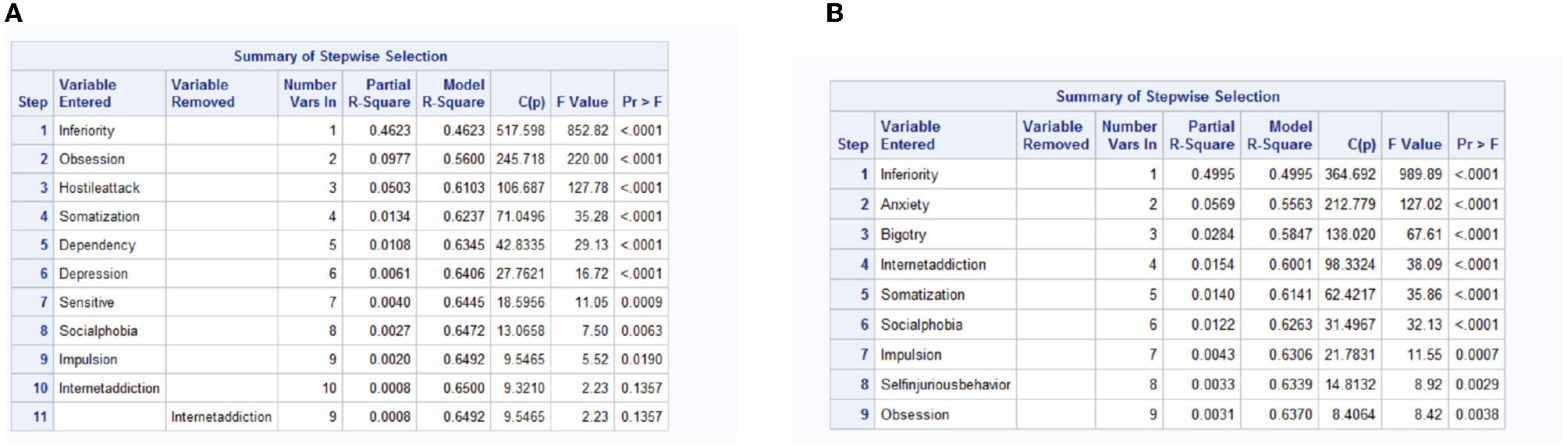
The effect factors for anxiety and depression for rural freshmen. **(A)** Anxiety. **(B)** Depression.

**Figure 4:**
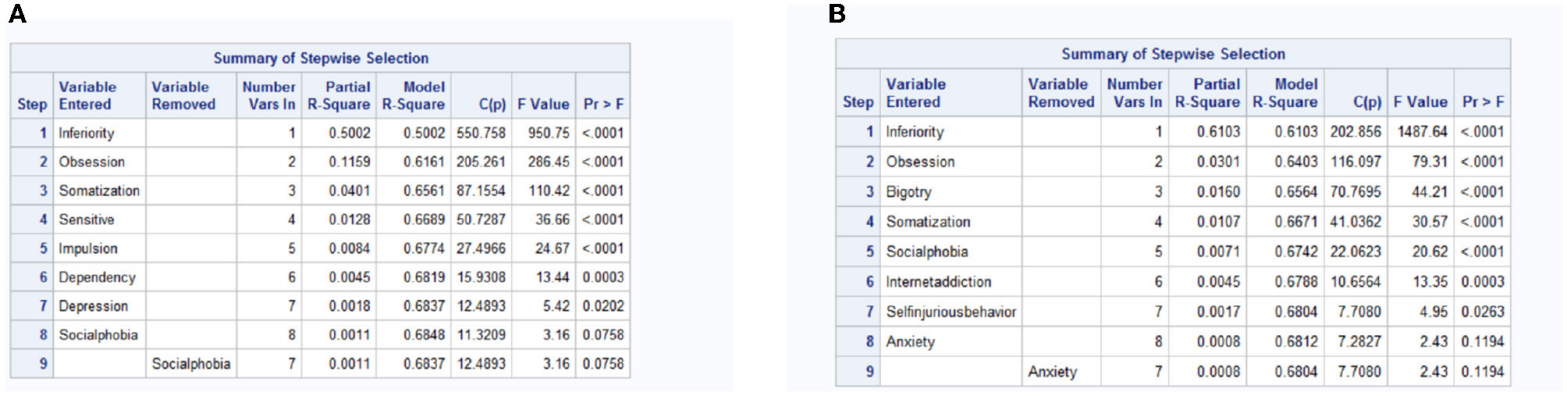
The effect factors for anxiety and depression for urban freshmen. **(A)** Anxiety. **(B)** Depression.

**Figure 5:**
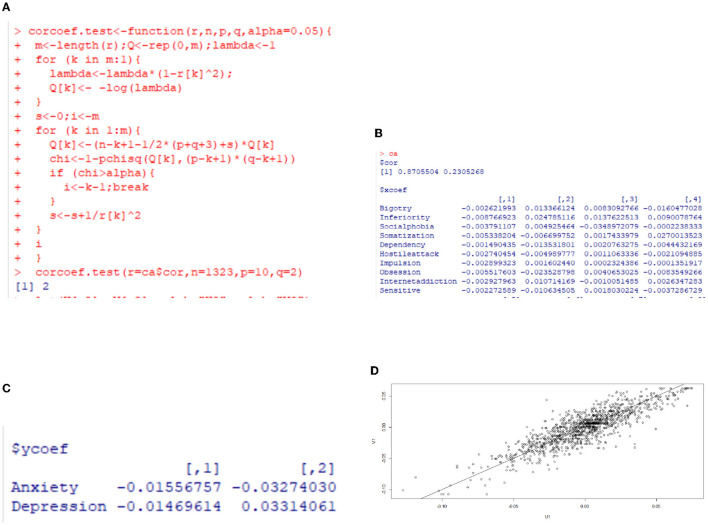
Canonical correlation analysis for anxiety and depression for rural freshmen. **(A)** Significance test of canonical correlation coefficient. **(B)** Correlation coefficient. **(C)** Correlation coefficient. **(D)** Score plane isogram.

**Figure 6:**
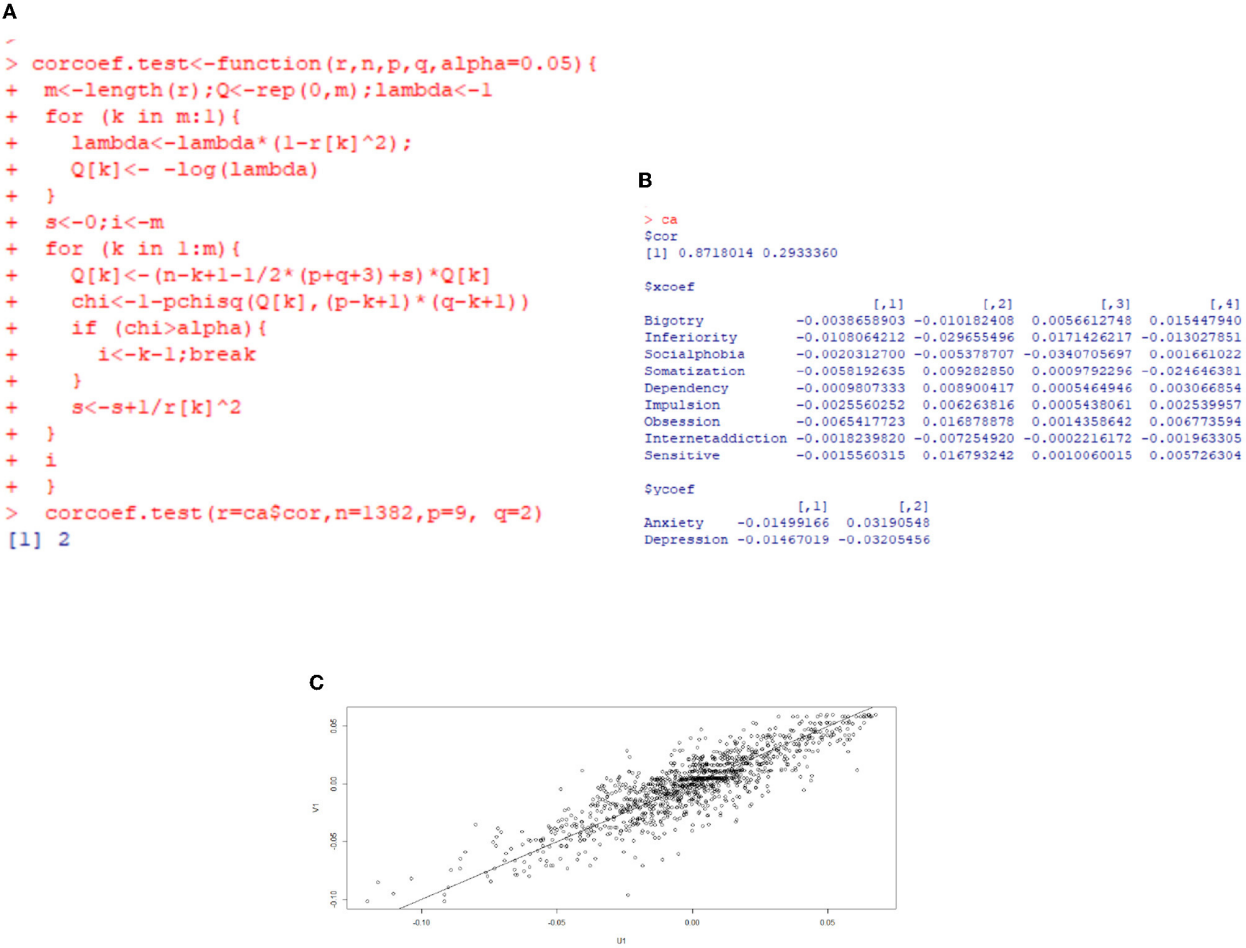
Canonical correlation analysis for anxiety and depression for urban freshmen. **(A)** Significance test of canonical correlation coefficient. **(B)** Correlation coefficient. **(C)** Score plane isogram.

To obtain the significant influencing factors for anxiety and depression of rural and urban freshmen, we take linear regression with stepwise selection separately for them. The results are in Figures 3, 4. Based on the significant effect factors (effect factors with *P* value < 0.05) in the results, the canonical correlation analysis is processed, and the results for rural and urban freshmen are in Figures 5, 6.

Figure 5 is the results of canonical correlation analysis for rural freshmen. The correlation coefficient for the first pair of variables is 0.8705504 (in Figure 5B), indicating a strong correlation, whereas the correlation coefficient for the second pair is 0.2305268, suggesting a relatively weak correlation. Additionally, based on the significance test for the canonical correlation coefficient in Figure 5A, it is sufficient to analyze only the first pair of canonical variables. The coefficients of variables are summarized in Table 5.

**Table 5:** Coefficients of variables for rural freshmen.

**Variable**	**Coefficient**
Bigotry	–0.002621993
Inferiority	–0.008766923
Social phobia	–0.003791107
Somatization	–0.005338204
Dependency	–0.001490435
Hostile attack	–0.002740454
Impulsion	–0.002899323
Obsession	–0.005517603
Internet addiction	–0.002927963
Sensitive	–0.002272589
Anxiety	–0.01556757
Depression	–0.01469614

Denote the effect factors in Table 5 in sequence as *x*_1_ through *x*_10_, and anxiety and depression as *y*_1_ and *y*_2_. The first set of canonical correlation variables is expressed by:


(2) $$\{\begin{array}{l} U=-0.002621993x_{1}-0.008766923x_{2}-0.003791107x_{3}\\ -0.005338204x_{4}-0.001490435x_{5}\\ -0.002740454x_{6}-0.002899323x_{7}-0.005517603x_{8}\\ -0.002927963x_{9}-0.002272589x_{10}\\ V=-0.01556757y_{1}-0.01469614y_{2} \end{array}$$

Here *U* is the linear combination of the significant influencing factors, and *V* is the linear combination of anxiety and depression.

By Bland (31) and Fei (30), the coefficient, which is also referred to as loading, of each component denotes the importance of this component.

The factors that have the greatest impact on anxiety and depression for rural freshmen, listed in order of their relatively larger loadings, are *x*_2_, *x*_8_, *x*_4_, and *x*_3_, indicating that inferiority, obsession, somatization, and social phobia are the primary factors. The moderate loadings of *x*_9_ and *x*_7_ suggest that internet addiction and impulsion also play a significant role. All of these factors have a positive effect on anxiety and depression.

Based on the isogram of the score plane depicted in Figure 5C, the data points are approximately aligned along a straight line. This indicates that the correlation between the first pair of canonical correlation variables can be effectively explained through the analysis, and this correlation is consistent and reliable.

Figure 6 is the results of canonical correlation analysis for urban freshmen. The correlation coefficient for the first pair of variables is 0.8718014, indicating a strong correlation, whereas the correlation coefficient for the second pair is 0.2933360, suggesting a relatively weak correlation. Additionally, based on the significance test for the canonical correlation coefficient in Figure 6A, it is sufficient to analyze only the first pair of canonical variables. The coefficients of variables are summarized in Table 6.

**Table 6:** Coefficients of variables for urban freshmen.

**Variable**	**Coefficient**
Bigotry	–0.0038658903
Inferiority	–0.0108064212
Social phobia	–0.0020312700
Somatization	–0.0058192635
Dependency	–0.0009807333
Impulsion	–0.0025560252
Obsession	–0.0065417723
Internet addiction	–0.0018239820
Sensitive	–0.0015560315
Anxiety	–0.01499166
Depression	–0.01467019

Denote the effect factors in Table 6 in sequence as *x*_1_ through *x*_9_, and anxiety and depression as *y*_1_ and *y*_2_. The expression of the first pair of canonical correlation variables is:


(3) $$\{\begin{array}{l} U=-0.0038658903x_{1}-0.0108064212x_{2}-0.0020312700x_{3}\\ -0.0058192635x_{4}-0.0009807333x_{5}\\ -0.0025560252x_{6}-0.0065417723x_{7}-0.0018239820x_{8}\\ -0.0015560315x_{9}\\ V=-0.01499166y_{1}-0.01467019y_{2} \end{array}$$

According to the formula, the primary impact variables for anxiety and depression for urban freshmen in sequence are inferiority (*x*_2_), obsession (*x*_7_), somatization (*x*_4_), and bigotry (*x*_1_). Impulsion (*x*_6_) and social phobia (*x*_3_) are moderate influencing factors.

Based on the isogram of the score plane depicted in Figure 6C, the data points are approximately aligned along a straight line. This indicates that the correlation between the first pair of canonical correlation variables can be effectively explained through the analysis, and this correlation is consistent and reliable.

### 3.4 The results for path analysis

#### 3.4.1 Path analysis for effect factors of depression (mediated by anxiety) for rural freshmen

Denote anxiety and depression as *Y*_1_ and *Y*_2_. For rural freshmen, denote inferiority, social phobia, obsession, somatization and internet addiction as *X*_1_, *X*_2_, *X*_3_, *X*_4_, *X*_5_, respectively. The strength of the relationship between each effect factor and depression is measured by effect size (also known as effect value).

**Figure 7:**
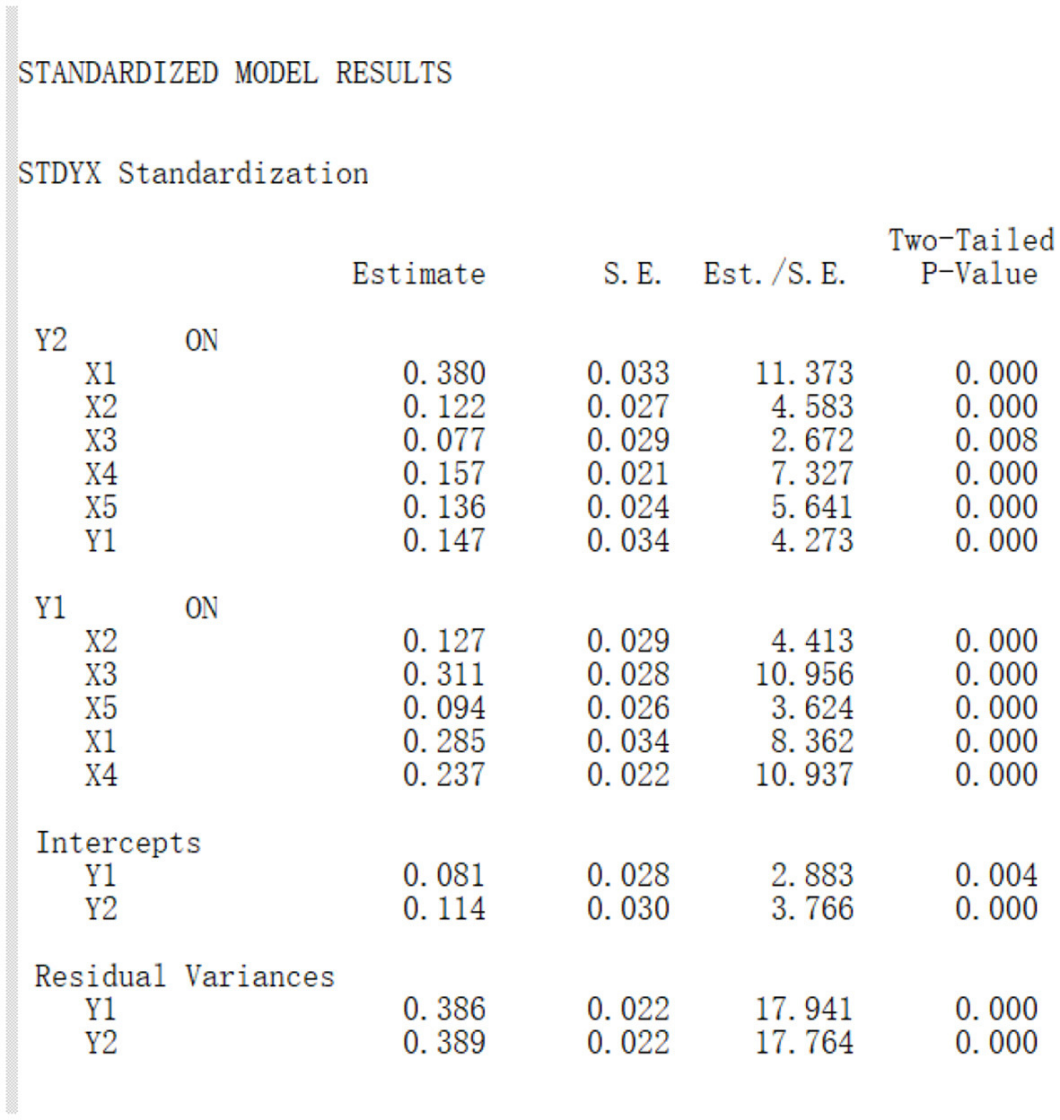
Path analysis for effect factors of depression (mediated by anxiety) for rural freshmen.

The Mplus result for path analysis for influencing factors of depression (mediated by anxiety) for rural freshmen is in Figure 7. For this model, CFI = 0.982, TLI = 0.971, Chi-Square Test = 2,506.108, and degrees of Freedom = 11, which means the model fits well. From *P* values in Figure 7, all of the direct and indirect pathes are significant on 0.01 level, and all estimates of standardized effect sizes are positive. This means anxiety has significant mediating effect on the relationship between the effect factors and depression, and all effect factors in the model have significant positive predictive effect for depression.

Based on the results, we we drew the path diagram on below, and conducted the effect decomposition of the effect factors of depression (mediated by anxiety) in Table 7.

**Table 7:** Effect decomposition for the effect factors of depression for rural freshmen.

**Effect**	**Direct path**	**Direct effect size**	**Indirect path**	**Indirect effect size**	**Total effect size**
*X*_1_ to *Y*_2_	*X*_1_→*Y*_2_	0.380	*X*_1_→*Y*_1_→*Y*_2_	0.042	0.422
*X*_2_ to *Y*_2_	*X*_2_→*Y*_2_	0.122	*X*_2_→*Y*_1_→*Y*_2_	0.019	0.141
*X*_3_ to *Y*_2_	*X*_3_→*Y*_2_	0.077	*X*_3_→*Y*_1_→*Y*_2_	0.046	0.123
*X*_4_ to *Y*_2_	*X*_4_→*Y*_2_	0.157	*X*_4_→*Y*_1_→*Y*_2_	0.035	0.192
*X*_5_ to *Y*_2_	*X*_5_→*Y*_2_	0.136	*X*_5_→*Y*_1_→*Y*_2_	0.014	0.150

Path diagram for effect factors of depression (mediated by anxiety) for rural freshmen:


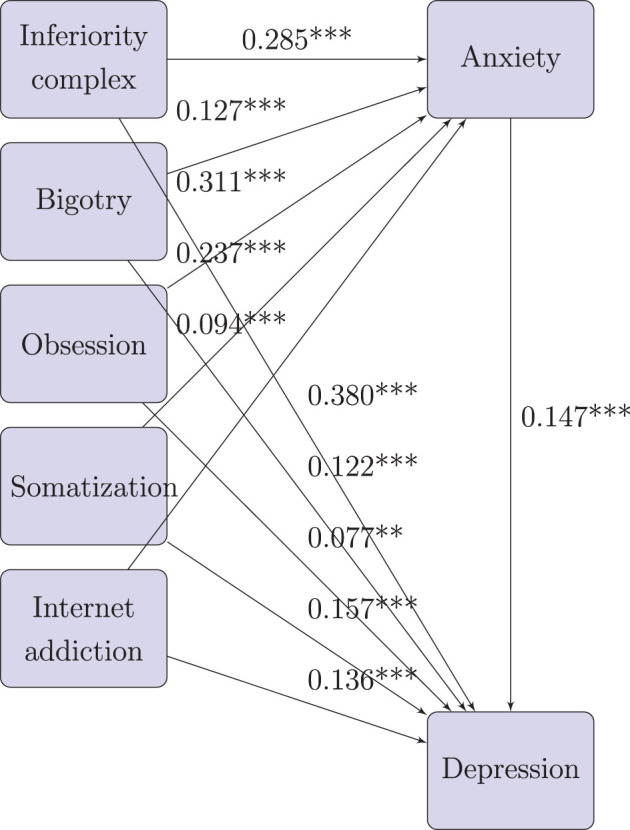


By Xiaoqun (34) and Hongyun (35), the indirect effect size of *X*_*i*_ to *Y*_2_ (*i* = 1…5) is computed by the effect size of *Y*_1_ on *X*_*i*_ times the effect size of *Y*_2_ on *Y*_1_. For example, the indirect effect size of *X*_1_ to *Y*_2_ is 0.285 × 0.147 = 0.042.

From Table 7, for rural freshmen, the effect size of inferiority (0.422) is much higher than other effect factors. The effect sizes of other effect factors in sequence are somatization (0.192), internet addiction (0.150), social phobia (0.141), and obsession (0.123).

#### 3.4.2 Path analysis for effect factors of depression (mediated by anxiety) for urban freshmen

For urban freshmen, denote inferiority, bigotry, obsession, somatization, and internet addiction as *X*_1_, *X*_2_, *X*_3_, *X*_4_, and *X*_5_, respectively.

**Figure 8:**
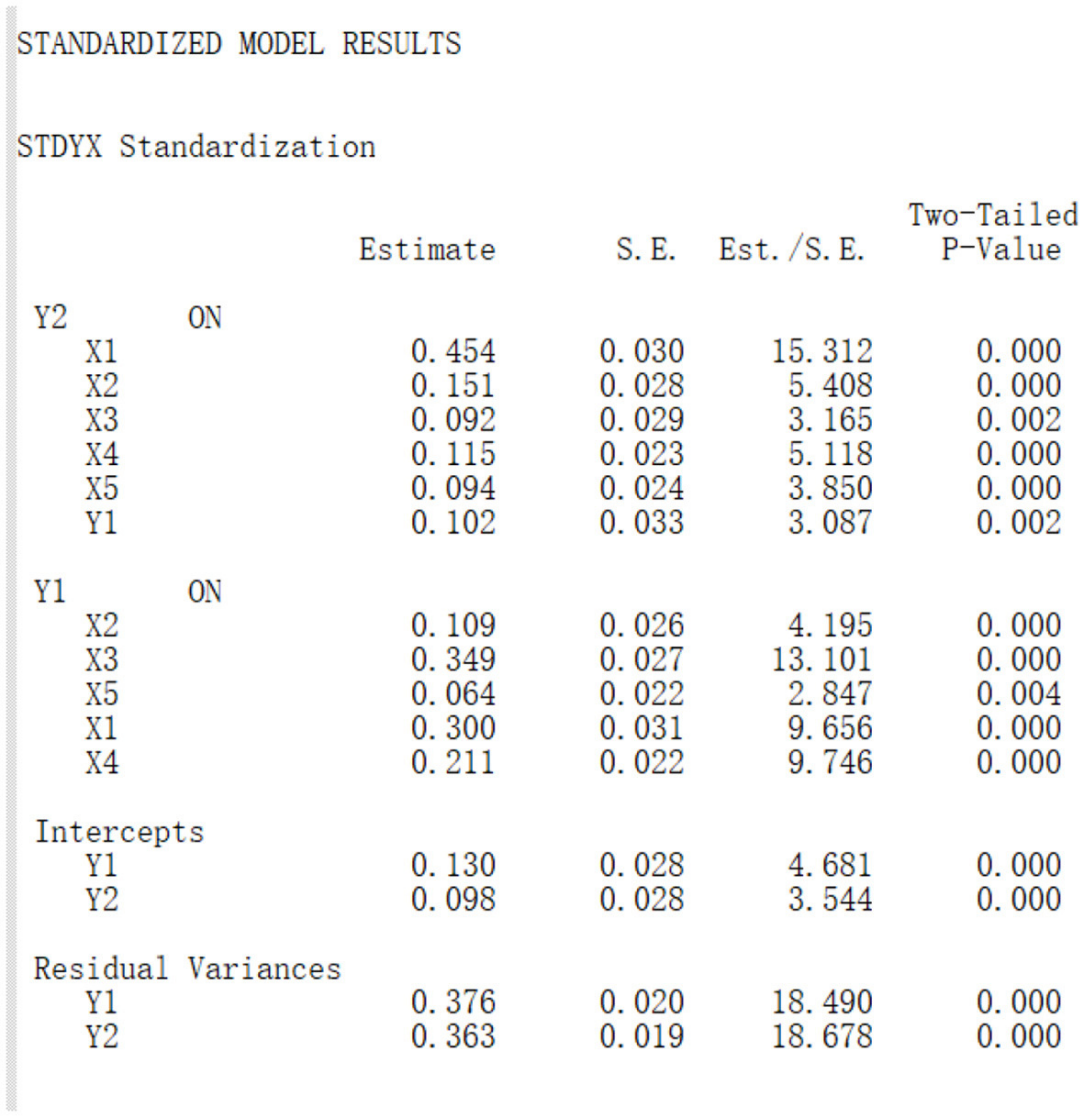
Path analysis for effect factors of depression (mediated by anxiety) for urban freshmen.

The Mplus result for path analysis of urban freshmen is in Figure 8. For this model, CFI = 0.991, TLI = 0.986, Chi-Square value = 2,751.772, and degrees of freedom = 11, which means the model fits well. From P values in Figure 8, all direct and indirect pathes are significant on 0.01 level, and all estimates of standardized effect sizes are positive.

Based on the results, we drew the path diagram on below, and conducted the effect decomposition of the effect factors of depression (mediated by anxiety) in Table 8.

**Table 8:** Effect decomposition for the effect factors of depression for urban freshmen.

**Effect**	**Direct path**	**Direct effect size**	**Indirect path**	**Indirect effect size**	**Total**
*X*_1_ to *Y*_2_	*X*_1_→*Y*_2_	0.454	*X*_1_→*Y*_1_→*Y*_2_	0.031	0.485
*X*_2_ to *Y*_2_	*X*_2_→*Y*_2_	0.151	*X*_2_→*Y*_1_→*Y*_2_	0.011	0.162
*X*_3_ to *Y*_2_	*X*_3_→*Y*_2_	0.092	*X*_3_→*Y*_1_→*Y*_2_	0.036	0.128
*X*_4_ to *Y*_2_	*X*_4_→*Y*_2_	0.115	*X*_4_→*Y*_1_→*Y*_2_	0.022	0.137
*X*_5_ to *Y*_2_	*X*_5_→*Y*_2_	0.094	*X*_5_→*Y*_1_→*Y*_2_	0.007	0.101

Path diagram for effect factors of depression (mediated by anxiety) for urban freshmen:


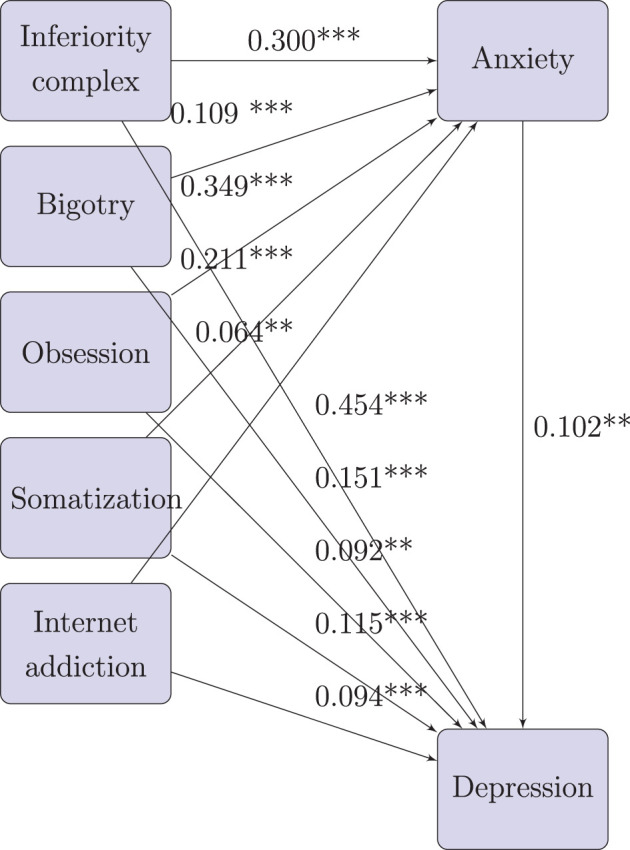


From Table 8, for urban freshmen, the effect size of inferiority (0.485) is much higher than other effect factors. the effect sizes of other effect factors in sequence are bigotry (0.162), somatization (0.137), obsession (0.128), and internet addiction (0.101).

## 4 Discussion and conclusion

From the investigation and analysis, levels of anxiety and depression of rural freshmen are significantly higher than that of urban freshmen. Inferiority, obsession, somatization, and internet addiction are the main effect factor for anxiety and depression of both rural and urban freshmen. For rural freshmen, social phobia is a noteworthy main effect factor for anxiety and depression, and for urban freshmen, bigotry is a specific main effect factor for anxiety and depression. Path analysis shows anxiety has significant mediating effect on the relationship between the main effect factors and depression. These results substantially extend former research in this area.

The manifestations of these factors includes many aspects. For rural freshmen, the inferiority and social phobia mainly manifest as a fear of being looked down upon, and a tendency to be less proactive in interpersonal interactions compared to urban freshmen. Jinling (17) and Yulong (18). For urban freshmen, the bigotry usually manifest as excessive sensitivity to setbacks and rejection, and overreactions in interpersonal relationships. For both rural and urban freshmen, the internet addiction mainly manifest as spending extensive hours online to escape the pressures of life (38).

These findings have important implications for designing practical intervention strategies. First, universities and policymakers should consider the establishment and enhancement of mental health support services on campuses. This includes increasing the availability of counseling centers, mental health professionals, and resources for students. Since anxiety and depression commonly occur together, mental health workers should assess and intervene of anxiety and depression as a whole. Considering the mediating effect of anxiety in the relationship between the effect factors and depression, interventions targeting anxiety management and coping skills can be beneficial. These strategies involve cognitive-behavioral therapy, mindfulness practices, and stress reduction techniques.

Second, for students with anxiety and depression, mental health institutions could assess markers of their inferiority, obsession, somatization and internet addiction, and then take certain measures to reduce extent of these factors. For example, for freshmen with internet addiction, interventions may include educational campaigns, counseling services, promoting alternative activities (such as sports activities), and providing family and social support.

Moreover, the policymakers and mental health workers should tailor intervention and education to the specific needs of rural and urban freshmen. For rural freshmen, addressing social phobia should be a main point, with interventions aimed at reducing social anxiety and enhancing social support networks. For urban freshmen, tackling bigotry should be a priority. Promoting inclusively and diversity within the university environment can be effective in reducing the impact of these factors on anxiety and depression.

While our research has revealed significant differences between urban and rural freshmen, we should recognize the inherent diversity and individual variations within each group. Each student's experience is shaped by a unique combination of personal, socioeconomic, and environmental factors. Thus it is imperative to acknowledge the nuanced nature of these disparities and take tailored interventions and support systems that consider the individuality of each student.

### 4.1 Strengths and limitations

Our sampling investigation collected the data in recent 5 years, and employed PPS sampling, the investigation period and sample size is also scientifically determined. These measures ensured the strong timeliness of the investigation and the representative of the sample to the statistical population. We have also conducted comprehensive statistical analysis for mental influencing factors of anxiety and depression, as well as the mediating effect of anxiety on the relationship between the main influencing factors and depression.

This study also has certain limitations. First, the data was based on self-reported questionnaires, thus the information obtained may has a certain degree of subjectivity. Second, this survey mainly utilized the Mental Health Screening Scale for college students in China, which primarily targets the mental health issues of first-year college students. Some other factors contributing to anxiety and depression may not be included in this scale.

## Data availability statement

The raw data supporting the conclusions of this article will be made available by the authors, without undue reservation.

## Ethics statement

Before commencing the questionnaire, the participants provided written informed consent online. The research procedures adhered to the American Association for Public Opinion Research (AAPOR) reporting guidelines and were approved by the Research Ethics Committee at Shandong University of Finance and Economics in China.

## Author contributions

CL wrote the manuscript. CL and BS collected the data. All authors contributed to the article and approved the submitted version.

## References

[1] Berney T Black S Checinski K Crome I Feinmann C Gowers S. The mental health of students in higher education. R College Psychiatr. (2003). Available online at: https://www.rcpsych.ac.uk/docs/default-source/improving-care/better-mh-policy/college-reports/college-report-cr166.pdf?sfvrsn=d5fa2c24_2

[2] Stewart-Brown S Evans J Patterson J Petersen S Doll H Balding J. The health of students in institutes of higher education: an important and neglected public health problem? J Public Health Med. (2000) 22:492–9. 10.1093/pubmed/22.4.492 11192277

[3] Tomoda A Mori K Kimura M Takahashi T Kitamura T. One-year prevalence and incidence of depression among first-year university students in Japan: a preliminary study. Psychiatry Clin Neurosci. (2010) 54:583–8. 10.1046/j.1440-1819.2000.00757.x 11043810

[4] Cheung K Tam KY Tsang MH Zhang LW Lit SW. Depression, anxiety and stress in different subgroups of first-year university students from 4-year cohort data. J Affect Disor. (2020) 274:41. 10.1016/j.jad.2020.05.041 32469820

[5] Wei L. Prevalence and related risk factors of anxiety and depression among chinese college freshmen. J Huazhong Univ Sci Technol. (2015) 35:822. 10.1007/s11596-015-1512-4 26670430

[6] Adlaf EM Gliksman L Demers A Newton-Taylor B. The prevalence of elevated psychological distress among Canadian undergraduates: findings from the 1998 Canadian Campus Survey. J American College Health. (2001) 50:67–72. 10.1080/07448480109596009 11590985

[7] Lee J Jeong HJ Kim S. Stress, anxiety, and depression among undergraduate students during the COVID-19 pandemic and their use of mental health services. Innov High Educ. (2021) 46:519–538. 10.1007/s10755-021-09552-y 33907351 PMC8062254

[8] Amir Hamzah NS Nik Farid ND Yahya A Chin C Su TT Rampal SRL. The prevalence and associated factors of depression, anxiety and stress of first year undergraduate students in a public higher learning institution in Malaysia. J Child Fam Stud. (2019) 28:3545–57. 10.1007/s10826-019-01537-y

[9] Liu X Cao X Gao W. Does low self-esteem predict anxiety among Chinese college students? Psychol Res Behav Manag. (2022) 15:1481–1487. 10.2147/PRBM.S361807 35719193 PMC9199909

[10] Liu XQ Guo YX Xu Y. Risk factors and digital interventions for anxiety disorders in college students: stakeholder perspectives. World J Clinical Cases. (2023) 11:1442–57. 10.12998/wjcc.v11.i7.1442 36926387 PMC10011984

[11] Liu XQ Guo YX Zhang WJ Gao WJ. Influencing factors, prediction and prevention of depression in college students: a literature review. World J Psychiatry. (2022) 12:860–73. 10.5498/wjp.v12.i7.860 36051603 PMC9331452

[12] Chen Y Luo P Chang T. Urbanization and the urban-rural income gap in China: a continuous wavelet coherency analysis. Sustainability. (2020) 12:8261. 10.3390/su12198261

[13] Zhong S Wang M Zhu Y Chen Z Huang X. Urban expansion and the urban-rural income gap: empirical evidence from China. Cities. (2022) 129:103831. 10.1016/j.cities.2022.103831

[14] Wu JX He LY. Urban-rural gap and poverty traps in China: a prefecture level analysis. Appl Econ. (2018) 50:3300–14. 10.1080/00036846.2017.1420890

[15] Yuemin Z. Investigation and research on mental health of freshmen. Contempor Educ Sci. (2012) 11:3.

[16] Xiurong T Qi Y Jin G. Analysis and countermeasures of psychological status of college students from urban and rural areas. High Med Educ China. (2008) 6:3–4. 10.3969/j.issn.1002-1701.2008.06.002

[17] Jinling G. Psychological problems and psychological health counseling education of rural freshmen. Jilin Educ. (2010) 8:1.

[18] Yulong Q. Comparative analysis of the mental health of college students from urban and rural areas. China's Public Health Manag. (2005) 21:2. 10.3969/j.issn.1001-9561.2005.04.038

[19] Zhixue W. Comparison of mental health status of rural and urban students. Hunan Middle School Phys Educ Front. (2009) 7:67–77.

[20] Zhang D. Changes in urban and rural college freshmen's mental health. Psychol Explor. (2015) 35:561–566.

[21] Wang BJ Wang ZF. Investigation of the mental health of urban and rural freshmen. Chinese J Health Educ. (2007) 12:924–925.

[22] Wenting G Zhiqiang Z. Research on the adaptation and psychological intervention of rural university freshmen. View Horizon. (2021) 2:1–2.

[23] Ross SM. Introductory Statistics. London: Academic Press. (2017). 10.1016/B978-0-12-804317-2.00031-X

[24] Wackerly D Mendenhall W Scheaffer RL. Mathematical Statistics with Applications. Boston, MA: Cengage Learning. (2014).

[25] Fang X Yuan X Wei HU Deng L Lin X. The development of college students mental health screening scale. Stud Psychol Behav. (2018) 16:111. 10.3969/j.issn.1672-0628.2018.01.015

[26] Pollack MH. Comorbid anxiety and depression. J Clin Psychiat. (2005) 66:22. 10.1038/432456a 16336033

[27] Belzer K Schneier FR. Comorbidity of anxiety and depressive disorders: issues in conceptualization, assessment, and treatment. J Psychiatr Pract. (2004) 10:296–306. 10.1097/00131746-200409000-00003 15361744

[28] Khansa W Haddad C Hallit R Akel M Obeid S Haddad G. Interaction between anxiety and depression on suicidal ideation, quality of life, and work productivity impairment: results from a representative sample of the Lebanese population. Perspect Psychiatr Care. (2020) 56:270–279. 10.1111/ppc.12423 31321788

[29] Cummings CM Caporino NE Kendall PC. Comorbidity of anxiety and depression in children and adolescents: 20 years after. Psychol Bull. (2014) 140:816. 10.1037/a0034733 24219155 PMC4006306

[30] Fei Y. Multivariate Statistical Analysis with R. Beijing: Renmin University of China Press. (2014).

[31] Bland M. An Introduction to Medical Statistics. Oxford: Oxford University Press. (2015).

[32] Manly BF Alberto JAN. Multivariate Statistical Methods: A Primer. New York, NY: Chapman and Hall CRC. (2016). 10.1201/9781315382135

[33] Mukhopadhyay P. Multivariate Statistical Analysis. Singapore: World Scientific Publishing Company. (2008). 10.1142/6744

[34] Xiaoqun H. Multivariate Statistical Analysis, Chapter 10. Beijing: Renmin University of China Press. (2015).

[35] Hongyun L. Advanced Statistics for Psychology, Chapter 9. Beijing: Renmin University of China Press. (2019).

[36] Li G Hou G Yang D Jian H Wang W. Relationship between anxiety, depression, sex, obesity, and internet addiction in Chinese adolescents: a short-term longitudinal study. Addict Behav. (2019) 90:421–7. 10.1016/j.addbeh.2018.12.009 30553156

[37] Servidio R Bartolo MG Palermiti AL Costabile A. Fear of COVID-19, depression, anxiety, and their association with Internet addiction disorder in a sample of Italian students. J Affect Disor Rep. (2021) 4:100097. 10.1016/j.jadr.2021.100097

[38] Priego-Parra BA Triana-Romero A Pinto-Gálvez SM Ramos CD Salas-Nolasco O Reyes MM. Anxiety, depression, attitudes, and internet addiction during the initial phase of the 2019 coronavirus disease (COVID-19) epidemic: a cross-sectional study in Mexico. MedRxiv. 2020-05. (2020) 10.1101/2020.05.10.20095844.

[39] Moscovitch DA Hofmann SG Suvak MK In-Albon T. Mediation of changes in anxiety and depression during treatment of social phobia. J Consult Clin Psychol. (2005) 73:945–52. 10.1037/0022-006X.73.5.945 16287394

[40] Nima AA Rosenberg P Archer T Garcia D. Anxiety, affect, self-esteem, and stress: mediation and moderation effects on depression. PLoS ONE. (2013) 8:e73265. 10.1371/journal.pone.0073265 24039896 PMC3767811

